# Naproxen chemoprevention induces proliferation of cytotoxic lymphocytes in Lynch Syndrome colorectal mucosa

**DOI:** 10.3389/fimmu.2023.1162669

**Published:** 2023-05-03

**Authors:** Charles M. Bowen, Nan Deng, Laura Reyes-Uribe, Edwin Roger Parra, Pedro Rocha, Luisa M. Solis, Ignacio I. Wistuba, Valerie O. Sepeda, Lana Vornik, Marjorie Perloff, Eva Szabo, Asad Umar, Krishna M. Sinha, Powel H. Brown, Eduardo Vilar

**Affiliations:** ^1^ Department of Clinical Cancer Prevention, The University of Texas MD Anderson Cancer Center, Houston, TX, United States; ^2^ Translational Molecular Pathology, The University of Texas MD Anderson Cancer Center, Houston, TX, United States; ^3^ Division of Cancer Prevention, National Cancer Institute, Bethesda, MD, United States; ^4^ Clinical Cancer Genetics Program, The University of Texas MD Anderson Cancer Center, Houston, TX, United States

**Keywords:** Lynch Syndrome (hereditary nonpolyposis colorectal cancer), immunoprevention, image mass cytometry, bioinformactics, cancer prevention

## Abstract

**Background:**

Recent clinical trial data from Lynch Syndrome (LS) carriers demonstrated that naproxen administered for 6-months is a safe primary chemoprevention that promotes activation of different resident immune cell types without increasing lymphoid cellularity. While intriguing, the precise immune cell types enriched by naproxen remained unanswered. Here, we have utilized cutting-edge technology to elucidate the immune cell types activated by naproxen in mucosal tissue of LS patients.

**Methods:**

Normal colorectal mucosa samples (pre- and post-treatment) from a subset of patients enrolled in the randomized and placebo-controlled ‘Naproxen Study’ were obtained and subjected to a tissue microarray for image mass cytometry (IMC) analysis. IMC data was processed using tissue segmentation and functional markers to ascertain cell type abundance. Computational outputs were then used to quantitatively compare immune cell abundance in pre- and post-naproxen specimens.

**Results:**

Using data-driven exploration, unsupervised clustering identified four populations of immune cell types with statistically significant changes between treatment and control groups. These four populations collectively describe a unique cell population of proliferating lymphocytes within mucosal samples from LS patients exposed to naproxen.

**Conclusions:**

Our findings show that daily exposure of naproxen promotes T-cell proliferation in the colonic mucosa, which paves way for developing combination of immunoprevention strategies including naproxen for LS patients.

## Introduction

Lynch Syndrome (LS, MIM 120435), the most common hereditary colorectal cancer (CRC) syndrome, affects approximately one million people in the United States (1). The underlying molecular pathophysiology of LS has been leveraged as a model syndrome for understanding carcinogenesis in the setting of DNA mismatch repair (MMR) deficiency (2, 3). Despite surveillance guideline recommendations of annual colonoscopy for LS carriers, rapid interval tumors, inadequate compliance remain an unmet clinical challenge and compromising the survival of the LS patient population. Therefore, the aforementioned challenges pose the need for developing durable interventions such as immune- and chemo-preventive modalities.

Previous studies, such as the Colorectal Adenoma/Carcinoma Prevention Program-2 (CAPP-2) study found that the chronic use of aspirin, a non-steroidal anti-inflammatory drug (NSAID), had favorable efficacy as a chemoprevention for all LS-related tumors (4, 5). These studies established the framework for launching the ‘Naproxen Study’, a phase IB, placebo-controlled, randomized clinical trial (NCT02052908) in LS carriers (6). Results from this trial demonstrated that both high and low dose naproxen administered for 6-months is a safe primary chemoprevention that promotes activation of different resident immune cell types without increasing general lymphoid cellularity *via* reduction of proinflammatory prostaglandin levels. In addition, naproxen effectively modulated tumor growth while prolonging survival in a pre-clinical trial using a tissue-specific mouse model of LS (6).

At the time of publication of the original trial data, immune cell enrichment analysis based on mRNAseq data from normal colorectal mucosa using *in silico* cell deconvolution was not able to elucidate the resident immune cell types activated by naproxen, which remained an unanswered, yet highly intriguing question for improving future development of preventive measures in LS. Precisely discerning the types of activated immune cells upon naproxen administration was limited in the previous study, which did not incorporate tissue acquisition procedures for high-resolution single-cell omics, due to lack of technical approaches at the time of study implementation. However, in-house advancements of single-cell omics has afforded the ability to generate an integrative spatial profiles of immune cells within mucosal samples collected from a subset of available participant’s samples using image mass cytometry (IMC) and recently developed bioinformatic techniques (7–9). From these computational benchmarks, we have discovered that daily chemoprevention with naproxen at low (220 mg) and high (440 mg) dosing activates proliferation of resident cytotoxic lymphocytes in colonic mucosa, which opens new opportunities for refining immune-prevention strategies of patients with LS.

## Materials and methods

### Study design and participants

Patient samples used in this study come from participants enrolled to the original ‘Naproxen Study’. Participants provided written informed consent to participate in the study, and ethical approval was obtained from the Institutional Review Boards (IRB) of all participating centers (IRB MDACC 2013-0698 and PA12-0327). Details on the trial design and participant demographics have been already reported (6). Here, we present the sub-analysis of data only from participants enrolled to the trial at The University of Texas MD Anderson Cancer Center, one of the four trial sites.

### Tissue micro array

A total of 36 Formalin-fixed Paraffin-embedded (FFPE) colon biopsies from Lynch Syndrome participants (18 pre-treatment and 18 post-treatment tissue samples) were collected. Three unstained slides (4 µm thickness) sections were obtained from each sample to perform hematoxylin and eosin (H&E) and immunohistochemistry (IHC) staining. A pathologist (L.S.) evaluated H&E slides to determine the quality of the sample, selection of areas for tissue microarray (TMA) construction, and histomorphometry analysis. Two biopsy blocks were excluded due to artifacts and insufficient tissue for analysis. One single TMA block was created using up to two tissue cores (1 mm diameter) from each colon biopsy specimen for further Image Mass Cytometry (IMC) staining. Normal and neoplastic control tissues (appendix, lymph node, tonsil, spleen, placenta, breast carcinoma, and ovary carcinoma) for IMC staining were included in the TMA of colon biopsies block to evaluate the staining quality from each immune marker.

### Image mass cytometry staining

An Immuno-Oncology (IO)-IMC panel with 36 validated markers was designed to include lymphoid (CD45RO, CD3, CD4, CD8, CD19, Granzyme B, CD94, OX40), myeloid (CD11b, CD14, CD68, MPO, CD11c, HLA-DR, CD163, CD33), immune-modulatory (CCR6, PD-L1, CD73, FOXP3, TIM3, LAG3, VISTA, B7-H3, ICOS), epithelial (pan cytokeratin, panCK), proliferative (Ki67), and leukocyte/constitutive (CD45, β2-microglobulin, GAPDH, NaKATPase) markers. The IO-IMC panel was optimized by single plex-IHC assays of BSA-free monoclonal antibodies, followed by single plex indirect immunofluorescence (IF) assays of each metal-isotope-tagged antibody (Maxpar Labeling Kits; Fluidigm), and a final multiplex staining step containing the whole cocktail with 36 metal-isotope-tagged antibodies. A control TMA (4 μm diameter) including normal and neoplastic tissues (tonsil, appendix, placenta, prostate, breast carcinoma, ovarian carcinoma, and endometrioid carcinoma) was used for each staining assay, and to select the optimal concentration per antibody showing a constant cellular subtype expression, and a stable pattern of staining in the same control tissue cores observed by single- and multiplex staining. Fifty consecutive 4 µm thickness unstained slides were obtained from the TMA block of colon biopsy specimens. To select the unstained slides for IMC staining, three slides at different levels (1, 25, and 50) were stained with H&E. The level 25 showed the highest number of cases (46) with at least one core evaluable, therefore a standardized IMC manual staining protocol was performed in three adjacent levels (22, 23, and 24). The staining protocol included one single antigen retrieval step (Tris buffer, pH 9.0, two-cycles of 10 min at 99°C), an overnight IMC panel cocktail incubation step at 4°C followed by Iridium and Ruthenium counterstaining. One field of view (FOV) per core (800 µm x 800 µm) was ablated and imaged by Hyperion Imaging System (Fluidigm) and MCD Viewer Software (v1.0.560.2, Fluidigm) for further digital image analysis.

### Image mass cytometry data processing

A TIFF-format image file for each mass channel was generated from the MCD file and then segmented into single cells using the ImcSegmentaionPipeline (https://github.com/BodenmillerGroup/ImcSegmentationPipeline) (10). Briefly, we generated a probability map by classifying pixels using Ilastik v.1.1.929 based on a combination of antibody staining to identify membranes, nuclei (single cells), and filter the background (**Table 1**). Then, these probability maps were segmented into single-cell object masks using CellProfiler (Version 3.1.9). The epithelium compartments were identified and segmented by combining NaKATPase-176Yb and panCK-174Yb signals into CellProfiler. Finally, the mean density of each marker was summarized at the single-cell level for future analysis.

**Table 1:** Immune mass cytometry metal-labeled antibodies.

N	Marker	Metal	Description	Clone	Vendor	Titration (1:X)	Cat#
1	CD45RO	139La	Memory T-cells	T200/797	Abcam	500	ab212786
2	CCR6	141Pr	Treg	EPR22259	Abcam	1000	ab243852
3	CD137	142Nd	Check point	BLR051F	Bethyl	25	A700-051.
4	ICOS	143Nd	Check point	D1K2T	CST	50	89601BF
5	PD-L1	144Nd(2X)	Check point	E1L3N	CST	50	13684BF
6	CD68	145Nd	Macrophage	EPR20545	Abcam	1000	ab227458
7	MPO	146Nd	Neutrophil	EPR20257	Abcam	2500	ab221847
8	PD-1	147Sm	Check point	EPR4877	Abcam	25	ab186928
9	CD11c	148Nd	Dendritic cell	EP1347Y	Abcam	2000	ab216655
10	CD73	149Sm	Check point	BLR054F	Bethyl	500	A700-54.
11	HLA-DR	150Nd	Antigen Presentation	EPR3692	Abcam	350	ab215985
12	CD163	151Eu	Macrophage	BLR087G	Bethyl	400	A700-087.
13	Granzyme B	152Sm	T-cell Activation	D6E9W	CST	1000	46890BF
14	CD11b	153Eu	Macrophage	EPR1344	Abcam	500	ab216445
15	CD14	154Sm	Macrophage	EPR3653	Abcam	1500	ab214438
16	FOXP3	155Gd	Treg	BLR034F	Bethyl	25	A700-034.
17	TIM3	156Gd	Checkpoint	D5D5R	CST	200	45208BF
18	CD66b	158Gd	Activated neutrophil	EPR7701	Abcam	100	ab249555
19	LAG3	159Tb	T-cell Activation	D2G4O	CST	200	15372BF
20	CD39	160Gd	Treg	EPR20461	Abcam	250	ab232573
21	IDO-1	161Dy	Treg	D5J4E	CST	25	86630BF
22	Ki-67	162Dy	Proliferation	D2H10	CST	200	9027BF
23	VISTA	163Dy	Checkpoint	D1L2G	CST	50	64953BF
24	β2-microglobulin	164Dy	Other, MHC-I	D8P1H	CST	200	12851BF
25	CD19	165Ho	B-cells	D4V4B	CST	1500	90176BF
26	CD8a	166Er	CD8 cells	D8A8Y	CST	100	85336BF
27	CD33	167Er	Myeloid lineage	SP266	Abcam	600	ab238784
28	B7-H3	168Er	Check point	D9M2L	CST	25	14058BF
29	CD45	169Tm	Lymphocytes	EP322Y	Abcam	800	ab214437
30	CD94	170Er	NK cell/cytotoxic lymphocytes	EPR21003	Abcam	50	ab238166
31	OX40	171Yb	T-cell Activation	E9U7O	CST	25	61637BF
32	CD3e	172Yb	Lymphocytes	D7A6E	CST	100	85061BF
33	CD4	173Yb	T-helper Lymphocyte	EPR6855	Abcam	1000	ab181724
34	Cytokeratin	174Yb	Epithelial	AE1/AE3	Abcam	25	ab80826
35	GAPDH	175Lu	Other, control	14C10	CST	5000	2118BF
36	NaKATPase	176Yb	Other, control	EP1845Y	Abcam	200	ab167390

Thirty-six validated markers designed to include lymphoid, myeloid, immune-modulatory, epithelial, proliferative, and leukocyte/constitutive.

'#' is a short-hand abbreviation for number.

### Data normalization and processing

Regarding gating analysis, no data transformation or normalization was applied. Additionally, all data were analyzed as a single batch, and thus, batch correction was not required. The Centered log-ratio (CLR) method implemented in the R package Seurat (Version 4.1.1) (8) was used for UMAP visualization. To visualize the data, we performed the Barnes-Hut implementation of t-distributed neighborhood embedding (tSNE) based on the markers and plotted this embedding in 2-dimensional space (perplexity, 50; number of iterations, 1,000).

### Clustering

Single cells from every field of view (FOV) were combined and clustered by the PhenoGraph algorithm (9) using R package Rphenograph (Version 0.99.1.9003) by their mean marker correlations. The UMAP algorithm was used for visualization of the data. Then, cell populations of interest were manually named based on the expression of markers, and their relative abundance was calculated as the number of a given cell type of interest divided by the total number of cells in each compartment.

### Statistical analysis

Changes in the cluster proportions relative to the total number of cells in the epithelial and non-epithelial compartment were calculated. Wilcoxon tests were performed using the R (Version 4.0.5) base package. Bonferroni multiple testing adjustment was applied as indicated in each analysis.

## Results

**Figure 1:**
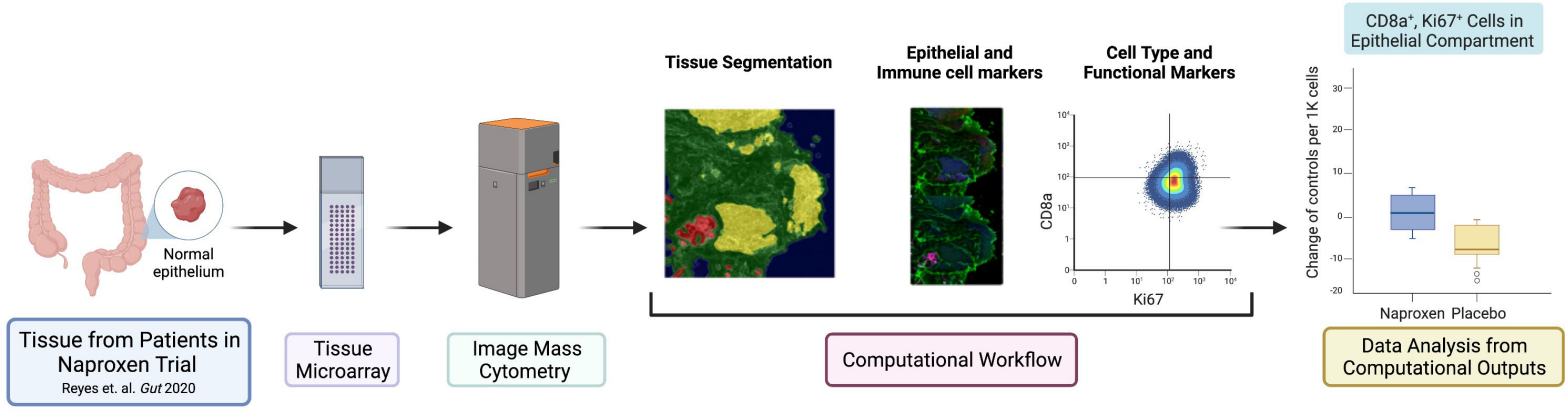
Graphical abstract of study design and workflow. The panel illustrates the schematic of experimental design and workflow used in this study. Normal rectosigmoid colon biopsies were collected from 18 LS participants [placebo N=6, low dose (LD, 220 mg) N=6, high dose (HD, 440 mg) N=6]. Participants (N=18) provided a pre- and post-exposure biopsy samples (N=36 samples) for tissue micro array (TMA), which was used for image mass cytometry (IMC) and analysis. This figure was generated using BioRender.com.

**Figure 2:**
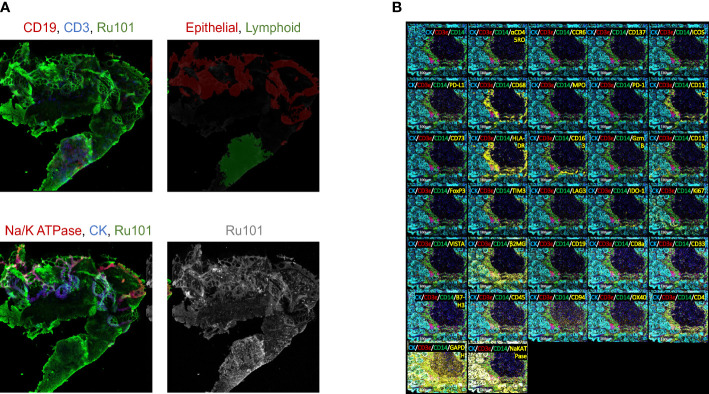
Immune mass cytometry isolated discrete immune cell populations following tissue segmentation. **(A)** Fluorescent image of tissue section stained for epithelial and immune cell markers. Upper left: red for CD19, blue for CD3, green for Ru101; Upper right: red for epithelial, green for lymphoid tissue; Bottom left: red for Na/K ATPase, blue for CK, green for Ru101; Bottom right shows Ru101; **(B)** Tissue section slides stained with multiplex metal-labeled antibody markers. Panel shows the representative image of IMC from an individual sample. Segmentation was performed to demarcate epithelium (cytokeratin, CK) from stroma. Metal-labeled antibody markers are listed in **Table 1**.

The experimental design and computational workflow are graphically described in **Figure 1** and detailed in the Materials and Methods section. Tissue biopsies from 18 patients (pre- and post-treatment; N=36 samples) enrolled in the ‘Naproxen Study’ were obtained for IMC analysis (**Supplementary Tables 1**, **2**). Tissue segmentation helped segregate the epithelial compartment from the stroma utilizing a multitude of markers for lymphoid (i.e. CD19, CD3) and epithelium (i.e. pan cytokeratin, panCK), which shows clear delineation between the two cellular compartments within the biopsy samples (**Figure 2A** and **Table 1**). Then, tissue sections were stained with 36 different metal-isotope-tagged antibodies (**Table 1**) to identify the localization of epithelial and immune cell types (**Figure 2B**) within the epithelial compartment previously identified from the segmentation analysis.

**Figure 3:**
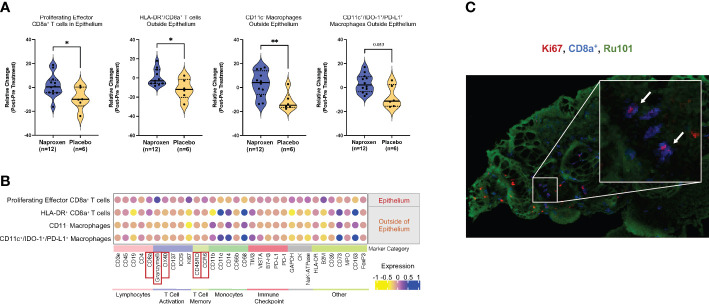
Chemopreventive naproxen increases activation of cytotoxic T-lymphocytes in the mucosa. **(A)** Data-driven analysis of the cell population within the mucosa. Change in cell ratio in those immune cell types for the naproxen and placebo groups that showed statistically significant changes. Solid line denotes median and dashed line denotes quartiles. *P-*values from paired Wilcoxon test from left to right: *P*=0.032, *P*=0.048, *P*=0.0047, *P*=0.052 (*, *P*-value<0.05; **, *P*-value<0.01); **(B)** Dot-plot graph shows the expression level of stained makers for the four cell types with statistically significant changes between naproxen and placebo groups. The color gradient represents the mean expression level of the corresponding marker in the population. Markers are grouped into categories based on cell type; Red boxes indicate expression markers of highlighted in the text; **(C)** High-resolution IMC image of Ki67^+^ (red), CD8a^+^ (blue), and Ru101 (green) in a LS mucosal sample. The white arrows indicate double-positive CD8+/Ki67+ cells in the colonic mucosa.

Using data-driven exploration, unsupervised clustering by the Phenograph algorithm generated clusters of 165 cell populations (9). From this output, we found four distinct immune cell populations of interest with statistically significantly changes between the treatment and control groups (**Figure 3A**; **Supplementary Figure 1A**). These cells are CD8a^+^ T cells in the epithelium, and immune cells that are outside of epithelium are HLA-DR^+^/CD8a^+^ T cells, CD11c- macrophages, and CD11c^+^/IDO-1^+^/PD-L1^+^. The absolute relative change between naproxen versus placebo for each group (**Figure 3A**, left to right) was 10.74 ± 4.73, 12.10 ± 4.77, 14.62 ± 4.91, and 11.17 ± 4.06, respectively, suggesting sustained cellularity of mucosal resident immune cell types by chronic use of naproxen in LS patients. To elucidate if this sustained observation was dose-dependent, we then analyzed the data by separating the naproxen groups (low-dose, 220 mg versus high-dose, 440 mg) and determined no significant difference in relative cellular abundance when comparing low-dose to high-dose groups (**Supplemental Figures 1B, C**). Furthermore, the function of each cell population was elucidated by quantifying the expression of gene markers related to lymphocyte and monocyte cellular markers (**Figure 3B**) that identified six different groups representing lymphocytes, T cell activation, T cell memory, monocyte, immune checkpoint and others. In accordance with our manual gating analysis, the levels of epithelial Ki67^+^/CD8a^+^/CD3e^+^ T cell population remained the same in the treatment group, but not in the placebo group, which showed an absolute reduction in levels over the course of the trial (*P-*value=0.032, **Figures 3A, B**; **Supplementary Figure 1A**). The relatively higher levels of CD45RO/CCR6 in the naproxen treatment than control group are indicative of an enrichment of T cell memory phenotype (11). Interestingly, activated memory T-cells correlated with high levels of OX40 (12), CD137 (13) and Granzyme B (14) expression (**Figure 3B**; **Supplementary Figures 2, 3**), thereby suggesting activation of T-cell function in anti-tumor immunity. High resolution IMC histology thus corroborated computational-based deconvolution findings, showing proliferating lymphocytes (CD8^+^/Ki67^+^) within mucosal samples from LS patients exposed to naproxen as visualized by the co-localization of Ki67 and CD8 staining (**Figure 3C**).

## Discussion

We have identified that proliferating (Ki67^+^), activated (OX40^+^/GZMB^+^/CD137^+^), memory (CD45RO/CCR6^+^) CD8a^+^ T-cells become activated upon naproxen treatment (12, 15). The importance of CD8^+^ T-cells in CRC biology has been well addressed in previously reported studies (16, 17) with a positive association between CD8a^+^ T-cell infiltration and favorable clinical outcomes (18). The stability in the numbers of activated CD8a^+^ T cells observed in our sub-cohort could be attributed to the reduction of prostaglandin E2 (PGE2) levels after using naproxen, thus confirming that reduction in PGE2 levels by naproxen has general anti-inflammatory effects, while promoting T cell retention in the colorectal mucosa (6). In addition to local effects of naproxen in the colonic mucosa, previous studies have shown elevated PGE2 levels in patient serum suppresses naïve memory and effector T cells with selective suppression of effector functions (19). In the original ‘Naproxen Trial’, global reduction in PGE2 levels correlated with activation of cytokine-related pathways and overall immune activation especially in B- and T-cell lineages (6). While the data reported in this manuscript explored the intrinsic local changes in immune activation within the colonic mucosa, it is possible that the effects of naproxen to peripheral immune cells contributed to the activation of immune cells within the mucosa of LS patients. Thus, the sustained immune cellularity after naproxen treatment is likely maintained by reduction of PGE2 levels at the peripheral and tissue-specific levels.

While an overall sustained immune response was observed for most trial participants, some of them demonstrated insufficient maintenance of immune cellularity despite receiving naproxen treatment (**Supplemental Figure 1C**). While the molecular mechanisms at play are beyond the scope of this manuscript, nuanced differences in the tissue microenvironment has been hypothesized to be the cause of immune evasion in some LS carriers (20), and therefore naproxen could help overcome it by exerting stimulation of the resident immune cells in the colorectal mucosa. Furthermore, the heterogeneity in lifelong neoantigen priming (21), known as ‘self-education’ of the immune system, is variable from person to person, and may partially help explain the lack of response (i.e. observed decrease in immune cellularity) to naproxen in some of the LS participants. Similarly, through self-priming mechanisms, it is also possible that LS patients experience T-cell anergy, which occurs along a continuum depending on relative neoantigen abundance. Nonetheless, these hypotheses warrant further investigation.

Although the original ‘Naproxen Trial’ demonstrated a dose-dependent effect between low-dose and high-dose naproxen-mediated immune activation, this study showed no dose-dependent effect, which is most likely explained by the small size or random biological variation between patients. It is also possible that naproxen-mediated immune activation encompasses a broad therapeutic window, which warrants further studies to assess dose effect on T lymphocyte activation.

This study utilized image mass cytometry (IMC) to quantify relative abundance of immune cells in the colonic mucosa. This powerful technique enabled high-resolution multiplexing of metal-tagged antibodies to determine naproxen sustained T-lymphocyte cellularity within the mucosal compartment of LS patients. Unlike more traditional methods such as immunofluorescence, IMC affords quantification of 40 markers within 135 detection channels. Moreover, the use of metal-tagged antibodies reduces spectral overlap and background signaling, which are often confounding disadvantages of immunofluorescence. Another advantage to IMC over more conventional methods is the customizability of metal conjugation reagents and spatial scanning of multiple regions of interest on the same slide (22, 23). While IMC has powerful capabilities and advantages, the equipment cost, panel optimization, and complex computation skills are major barriers of use.

While this study has further identified the overall impactful role of naproxen within the normal colonic mucosa, mechanistic studies, which remain lacking, are necessary to better understanding the molecular underpinnings at play that govern immune activation and enrichment especially given the heterogeneity between different LS patients. The exploration of those studies is warranted for further studies, though it is an important and undetermined questions in the field.

In summary, this work deepens our understanding of the cellular composition within the mucosal epithelium of LS carriers upon administration of naproxen for chemoprevention. Despite a more stringent schedule of annual or bi-annual colonoscopy screenings, patients with LS still develop CRC for various reasons such as rapid interval tumors (6). The need for robust, durable preventive strategies remains a dire unmet need for the clinical management of hereditary CRC syndromes. Moving forward, this study helps pave the way for adaptative immunoprevention strategies for LS carriers combining neoantigen vaccines with naproxen as immune stimulant (24, 25).

## Data availability statement

The datasets presented in this study can be found in online repositories. The names of the repository/repositories and accession number(s) can be found below: https://www.ncbi.nlm.nih.gov/geo/, GSE144381. https://zenodo.org/record/7655637#.ZEf9PXbMI2x.

## Ethics statement

The studies involving human participants were reviewed and approved by Institutional Review Board of University of Texas MD Anderson Cancer Center. The patients/participants provided their written informed consent to participate in this study.

## Author contributions

EV has full access to all the data in the study and takes responsibility for the integrity and accuracy of the data analysis. EV conceived and supervised the study, and provided critical resources to perform the experiments, and wrote the manuscript. CB and KS analyzed the data, and wrote the manuscript. ND performed the IMC and bioinformatics analysis. LR-U provided assistance on the analysis and interpretation of the data, writing and editorial assistance. LS, EP, PR, and IW assisted with pathology interpretation, and IMC analysis. ES, LV, AU, MP, PB and EV provided supervision to the clinical study. VS, EV provided identification of study subjects and clinical information. All authors contributed to the article and approved the submitted version.
