# Supplementary figures and images for: Naproxen chemoprevention induces proliferation of cytotoxic lymphocytes in Lynch Syndrome colorectal mucosa
Source: Front Immunol. 2023 May 3;14:1162669. doi: 10.3389/fimmu.2023.1162669 (PMC10189148; doi:10.3389/fimmu.2023.1162669)

Supplemental Figure 1

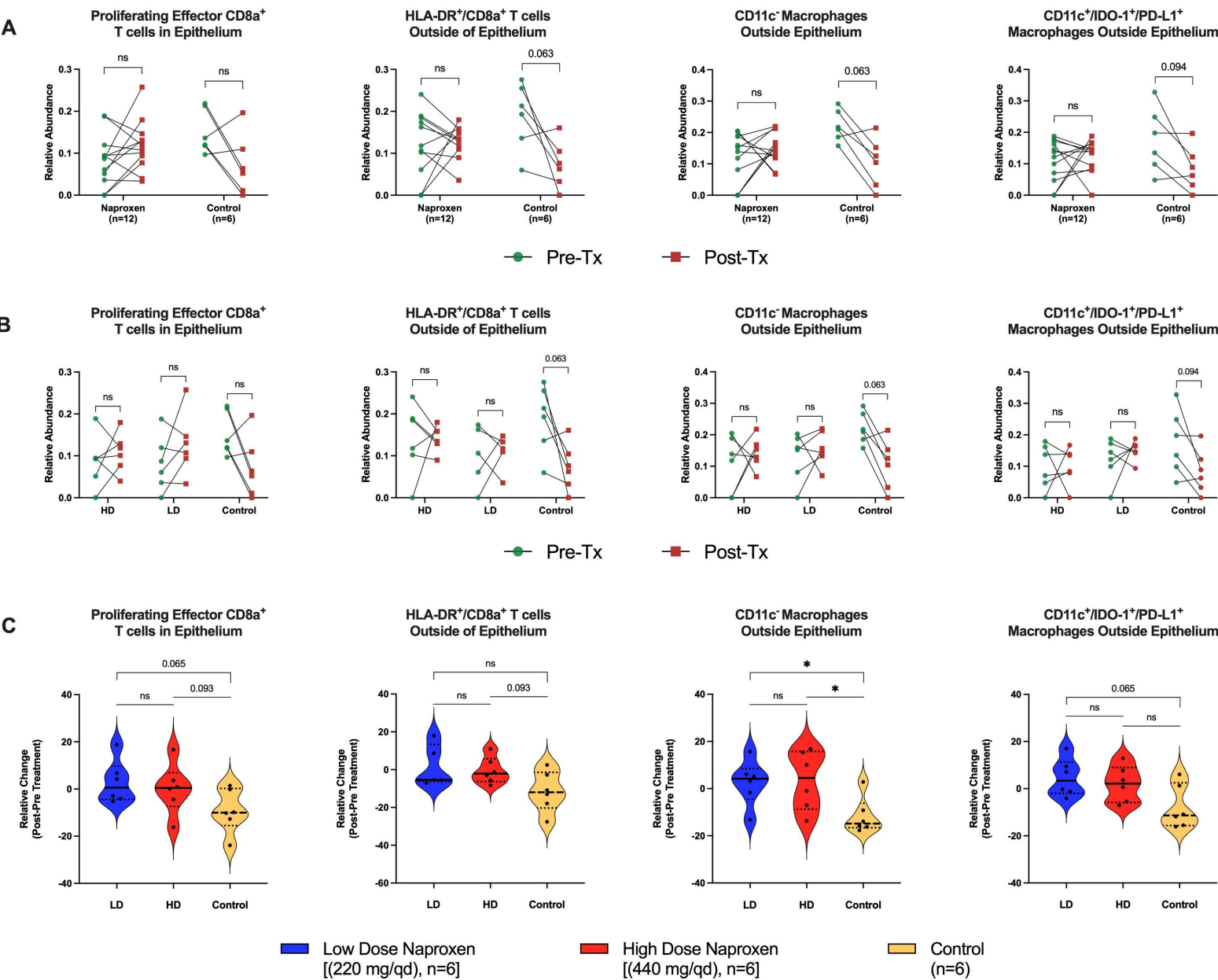

Supplemental Figure 2

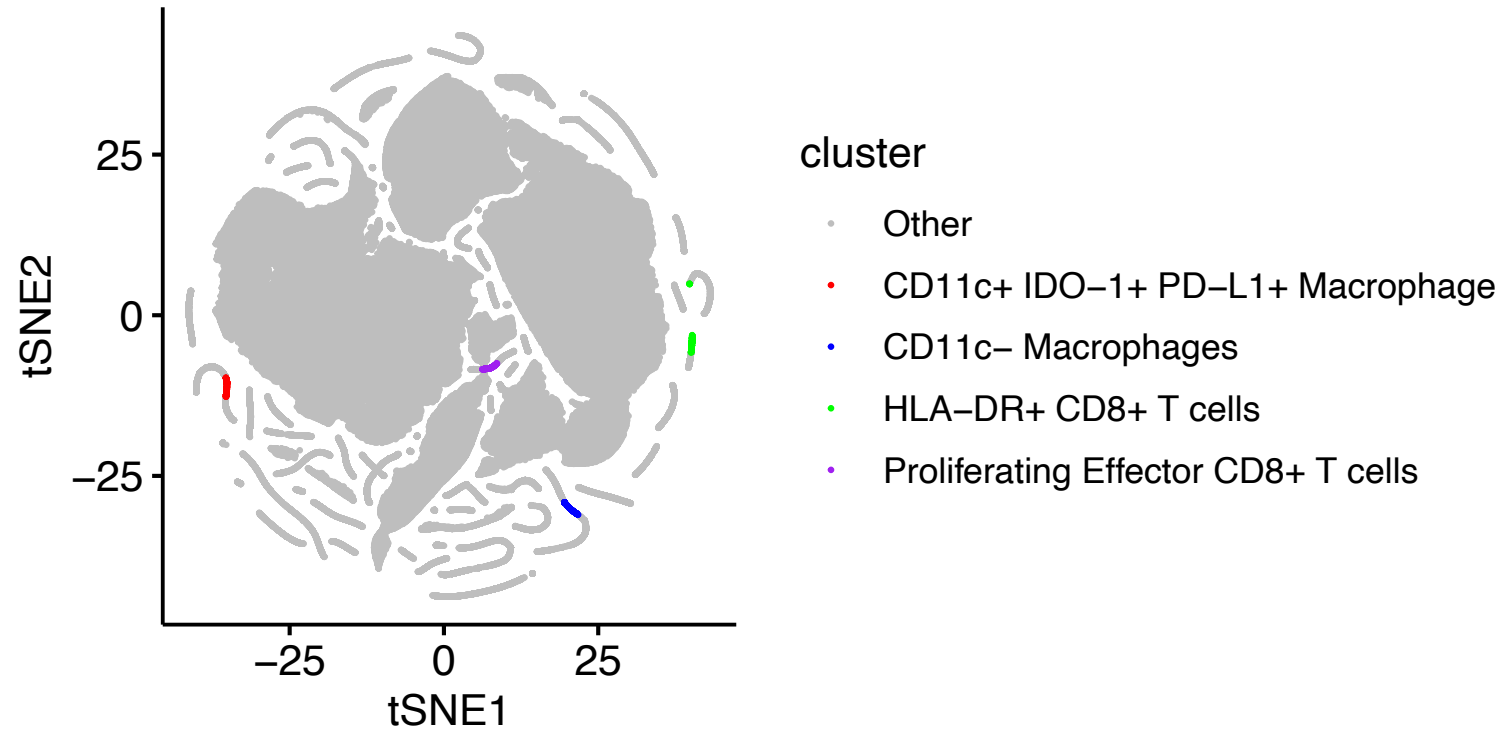

Supplemental Figure 3

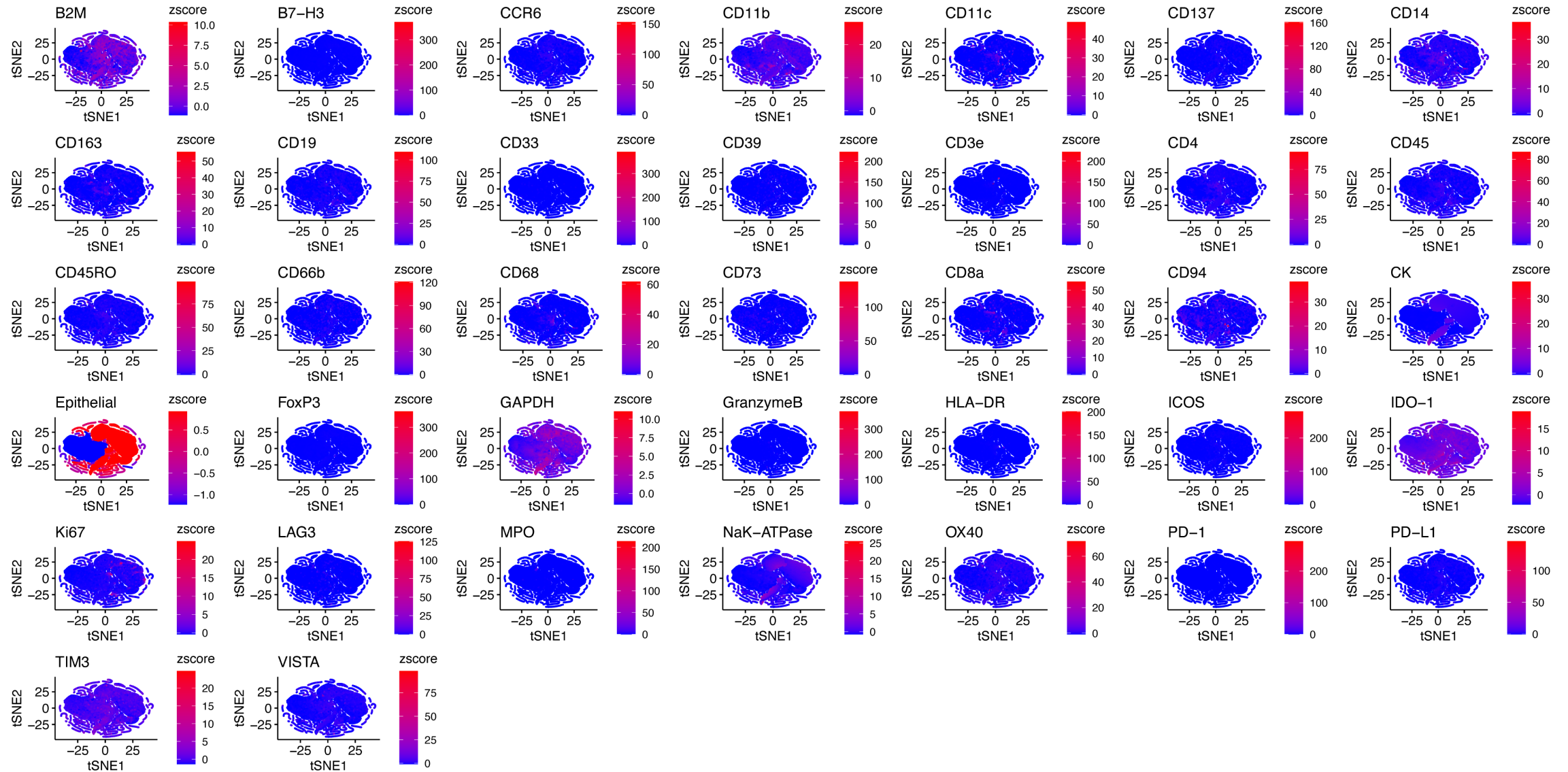

Supplement: Supplementary file 1 [file DataSheet_1.pdf]
