# Supplementary material for: Naproxen chemoprevention induces proliferation of cytotoxic lymphocytes in Lynch Syndrome colorectal mucosa
Source: Front Immunol. 2023 May 3;14:1162669. doi: 10.3389/fimmu.2023.1162669 (PMC10189148; doi:10.3389/fimmu.2023.1162669)
Supplement: Supplementary file 2 [file Table_1.docx]

**Supplemental Table 1.** Sample paring of participants. Each subject (N=18) had a pre- and post-treatment biopsy assessed (N=36 samples). Numbers of regions of interest (ROI) included in the analysis for each sample are listed.

| Subject | Treatment group | Number of ROIs  (pre-treatment) | Number of ROIs  (post-treatment) |
| --- | --- | --- | --- |
| 1 | High Dose Naproxen (440 mg/qd) | 1 | 2 |
| 2 | High Dose Naproxen (440 mg/qd) | 2 | 2 |
| 3 | High Dose Naproxen (440 mg/qd) | 2 | 1 |
| 4 | High Dose Naproxen (440 mg/qd) | 1 | 1 |
| 5 | High Dose Naproxen (440 mg/qd) | 2 | 1 |
| 6 | High Dose Naproxen (440 mg/qd) | 2 | 1 |
| 7 | Low Dose Naproxen (220 mg/qd) | 1 | 1 |
| 8 | Low Dose Naproxen (220 mg/qd) | 1 | 2 |
| 9 | Low Dose Naproxen (220 mg/qd) | 1 | 1 |
| 10 | Low Dose Naproxen (220 mg/qd) | 2 | 2 |
| 11 | Low Dose Naproxen (220 mg/qd) | 3 | 1 |
| 12 | Low Dose Naproxen (220 mg/qd) | 2 | 2 |
| 13 | Placebo | 2 | 2 |
| 14 | Placebo | 2 | 1 |
| 15 | Placebo | 2 | 1 |
| 16 | Placebo | 2 | 1 |
| 17 | Placebo | 1 | 1 |
| 18 | Placebo | 3 | 3 |
